# The effectiveness of problem solving therapy for stroke patients: study protocol for a pragmatic randomized controlled trial

**DOI:** 10.1186/1471-2377-13-67

**Published:** 2013-06-27

**Authors:** Marieke M Visser, Majanka H Heijenbrok-Kal, Adriaan van ’t Spijker, Gerard M Ribbers, Jan JV Busschbach

**Affiliations:** 1 Department of Rehabilitation Medicine, Erasmus MC, University Medical Center Rotterdam, PO Box 2040, 3000 CA, Rotterdam, the Netherlands; 2 Rijndam Rehabilitation Center, PO Box 23181, 3001 KD, Rotterdam, the Netherlands; 3 Department of Psychiatry, Erasmus MC, University Medical Center Rotterdam, Section Medical Psychology and Psychotherapy, PO Box 2040, 3000 CA, Rotterdam, the Netherlands

**Keywords:** Problem Solving Therapy, Stroke, Rehabilitation, Coping style, Health-related quality of life

## Abstract

**Background:**

Coping style is one of the determinants of health-related quality of life after stroke. Stroke patients make less use of active problem-oriented coping styles than other brain damaged patients. Coping styles can be influenced by means of intervention. The primary aim of this study is to investigate if Problem Solving Therapy is an effective group intervention for improving coping style and health-related quality of life in stroke patients. The secondary aim is to determine the effect of Problem Solving Therapy on depression, social participation, health care consumption, and to determine the cost-effectiveness of the intervention.

**Methods/design:**

We strive to include 200 stroke patients in the outpatient phase of rehabilitation treatment, using a multicenter pragmatic randomized controlled trial with one year follow-up. Patients in the intervention group will receive Problem Solving Therapy in addition to the standard rehabilitation program. The intervention will be provided in an open group design, with a continuous flow of patients. Primary outcome measures are coping style and health-related quality of life. Secondary outcome measures are depression, social participation, health care consumption, and the cost-effectiveness of the intervention.

**Discussion:**

We designed our study as close to the implementation in practice as possible, using a pragmatic randomized trial and open group design, to represent a realistic estimate of the effectiveness of the intervention. If effective, Problem Solving Therapy is an inexpensive, deliverable and sustainable group intervention for stroke rehabilitation programs.

**Trial registration:**

Nederlands Trial Register, NTR2509

## Background

Stroke is an increasing public health problem in the Netherlands: every year, 41,000 people suffer from stroke and over 3% of the total health care costs are related to the treatment of stroke and its consequences [1]. The mortality rate after stroke is 30% and is likely to decrease, which will cause an increase in morbidity [2]. Almost 50% of stroke survivors experience consequences in daily life that result in a lowered health-related quality of life (HR-QoL) [1]. The World Health Organization Quality of Life (WHOQOL) Group defines quality of life as “individuals’ perceptions of their position in life in the context of the culture and value systems in which they live and in relation to their goals, expectations, standards and concerns [3]. HR-QoL refers to the health-related aspects of quality of life. On average, utility scores of HR-QoL after stroke range from 0.47 to 0.68 (a utility score equal to death is 0.0 and full health 1.0), which is substantially lower than the value of a healthy reference population (utility score of 0.93) [4,5]. HR-QoL after stroke is predicted by functional constraints, age, gender, and psychosocial factors, like socioeconomic status, depression, and coping style [4,6-8]. Functional constraints, age, gender, and socioeconomic status cannot or are difficult to change, but coping style could be targeted. The question then becomes if HR-QoL after stroke could be improved through a coping style intervention. If this is possible, a secondary question would be how such improvement relates to depression, health care consumption, and costs.

A common definition of coping style is someone’s preferred way of dealing with different situations. Several coping styles can be distinguished, such as active, passive, and avoidant coping. Wolters (2010) shows that in traumatic brain-injured (TBI) patients, higher HR-QoL in the long term is predicted by an increase in active problem-focused coping style and a decrease in passive emotion-focused coping style. Unfortunately, in this population of TBI patients the active coping decreases over time, while passive coping increases [9]. This suggests that if the decrease of active coping can be stopped, there is room for improvement in HR-QoL. Stroke patients make even less use of active, problem-oriented coping styles compared to other brain damaged patients [10]. Furthermore, Darlington (2007) shows that in stroke patients, coping becomes more important in determining HR-QoL over time, while the importance of general functioning decreases [11]. This would mean that long term HR-QoL could benefit from improved coping.

Coping styles can be influenced by several interventions. Backhaus (2010) shows that an intervention aimed at changing maladaptive coping styles positively influenced psychosocial functioning of TBI patients [12]. However, HR-QoL was not measured in this study. No research is found that investigated an intervention aimed at improving HR-QoL through the change of maladaptive coping styles in stroke patients. We therefore set out to investigate whether Problem Solving Therapy (PST), which aims at active problem-focused coping, might improve HR-QoL in stroke patients. PST has been proved effective in other patient populations [13,14]. In stroke patients, PST has been shown successful for the prevention of post stroke depression [15]. Effects on coping style and HR-QoL have not been investigated yet.

### Objectives

The primary aim of this study is to investigate whether PST is an effective group intervention for improving active problem-focused coping style and HR-QoL in stroke patients. The secondary aim is to determine the effect of PST on depression, social participation, health care consumption, and the cost-effectiveness of the intervention. The effectiveness of the therapy will be investigated in an open group design, which has not been used in PST research before. PST will be added to the standard care just before the end of the rehabilitation program, as at this moment a relapse in HR-QoL is frequently observed, when patients cannot rely on their therapists anymore [16]. By teaching patients to actively cope with stressful situations, through adapting and realizing their goals, we expect that patients will use more effective coping styles, which consequently may prevent the relapse in HR-QoL, and possibly increase HR-QoL in the long term. With regard to the secondary aims of this study, we expect the incidence of depression to decrease, social participation to improve, and health care consumption to decrease, resulting in a favorable cost-effectiveness ratio for the intervention.

## Methods/design

### Study design and procedure

The effectiveness of PST for stroke patients will be evaluated in a multicenter pragmatic randomized controlled trial (RCT) with one year follow-up, with the intervention performed in the daily practice of a sub-acute outpatient stroke rehabilitation program. As such, the potential effects of the intervention have a good external validity, which allows us to calculate the cost-effectiveness of the therapy compared with standard care.

**Figure 1:**
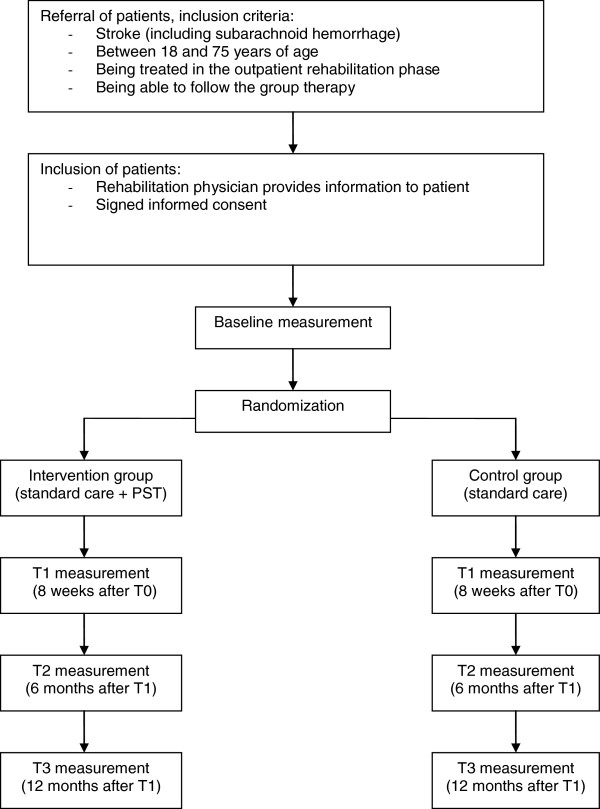
Design of the randomized controlled trial.

The study will be performed in Rijndam Rehabilitation Center in collaboration with Erasmus MC, both in the Netherlands, and in Ghent University Hospital in Belgium. Patients are invited by their rehabilitation physician to participate in the study. Before the start of the study, patients need to sign the informed consent form. Data will be collected at four time points by one of three research psychologists. T0 is the baseline measurement, performed within three weeks before the start of the intervention phase. T1 will be performed within ten days after the intervention phase, T2 six months and T3 twelve months after the intervention phase (Figure 1). The measurements will be performed in the rehabilitation center or at the patients’ home in a face-to-face interview. The study has been approved by the Medical Ethics Committee of Erasmus MC University Medical Center and the Ethics Committee of Ghent University Hospital.

### Study population

We strive to include 200 stroke patients. Inclusion criteria are: stroke (including subarachnoid hemorrhage), age between 18 and 75 years, being treated in the outpatient rehabilitation phase, and being able to participate in group therapy. Exclusion criteria are: progressive neurological disorders, life expectancy less than one year, insufficient understanding of the Dutch language, excessive drinking or drug abuse, subdural hematomas, moderate and severe aphasia. The same criteria would apply to the implementation of PST in practice, which stresses the pragmatic character of the trial. The inclusion of patients started March 2011 and will end August 2013. The one-year follow-up of all patients will be finished by September 2014.

### Randomization

Patients are randomized to the intervention- or control condition using a stratified block randomization procedure with a block size of four. To ensure comparability between the two groups, patients are stratified per rehabilitation center. A member of the research group, who is not involved in the collection of the data, prospectively allocates the patients to the intervention- or control condition in a one-to-one ratio using an online random-number generator. To allow blinded randomization, the allocation information will be put in separate sealed envelopes which are consecutively numbered. At the end of the baseline measurement, the investigator opens the numbered envelop and informs the patient about the condition he or she is assigned to. The research psychologists who perform the baseline and follow-up measurements are blinded for treatment condition. The therapists who provide the intervention are not involved in the collection of the data. The investigator who will analyze the data is not involved in the collection of the follow-up measurements.

### Intervention: problem solving therapy

Patients who are assigned to the intervention condition will receive PST in addition to the standard rehabilitation program, which will start during the last eight weeks of outpatient treatment. PST is a widely used and practical intervention method, based on a general model of coping with stress [17,18]. The model states that having a chronic disease causes stressful daily problems, which increase the chance of experiencing psychological stress and depressive feelings. Therefore, the aim of PST is to improve the skills to cope with the stressful daily problems in life after stroke.

**Figure 2:**
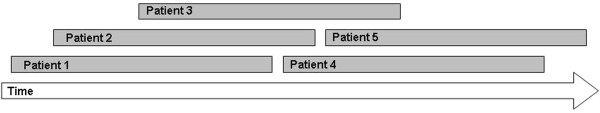
Patient flow in an open group therapy.

The intervention will be provided in an open group design, with a continuous flow of patients, which means that patients can enter the group every week and leave the group after eight sessions (Figure 2). The reason for this design is that it studies group therapy in its most feasible form, where patients start and end their programs at different time-points. If we had chosen to study the effect in closed groups, many patients in the similar stage of their programs are necessary. This would only be possible if patients are admitted to large scale rehabilitation centers, which is not the rehabilitation practice in The Netherlands, or patients would have to wait for a long time before entering the group. The open group design has some disadvantages. Patients may feel unsafe when they enter an already existing group. Furthermore, a continuous flow of patients is required to keep a balanced number of patients in the group. Therefore, interventions aimed at rare diseases cannot be studied with an open group design. However, for our population of stroke patients we do expect the design to be suitable and beneficial, because these patients are frequently seen in rehabilitation treatment. An open group design has several benefits as well. Advantages for the patients are that they do not have to wait until they can start with the intervention, they can share their experiences with other ‘experienced’ stroke patients, and there is room for interaction with many fellow patients. Other advantages are that the intervention is relatively easy to organize and implement in the daily practice of the rehabilitation center. This open group design has not been investigated in PST research yet.

The intervention in this study consists of eight group sessions of 1,5 hours a week, with homework exercises after each session. The group consists of a minimum of three and a maximum of six participants. PST is provided by one to three trained neuropsychologists per rehabilitation center. Solving problems will be structured, by dividing the problem solving process in four steps:

1. Define problem and goal;

2. Generating multiple solutions;

3. Considering the possible consequences of the solutions systematically and select the best solution;

4. Implement the solution and evaluate.

Each session starts with the sharing of experiences from the past week. Then, the model of problem solving will be repeated and explained. If there are some participants who are in the group for a couple of weeks already, they will be asked to explain the model to other new participants. Subsequently, one step of the model will be highlighted every week. With emphasis on this specific step, the model will be applied to one or more examples from the participants. Finally, the participants will be asked to practice the specific step at home by making a homework assignment. During the sessions, inadequate and irrational thoughts will be challenged by common cognitive interventions. A unique aspect of the intervention is the focus on the definition of the problem in the first step of the model. A clear definition of the problem will lead to a better understanding and more solutions to it.

### Control condition: standard care

Patients who are assigned to the control condition will receive the standard rehabilitation program, in order to be able to study the additional effect of the intervention to the standard rehabilitation program. This standard rehabilitation program consists of individualized amounts of treatment by a physical therapist, occupational therapist, speech therapist, psychologist, social worker, and rehabilitation physician, depending on the severity of stroke. On average, stroke patients in outpatient rehabilitation receive twelve hours of treatment a week during a nine week rehabilitation program.

### Outcomes

Primary outcome measures are changes in task-oriented coping and psychosocial HR-QoL in patients in the intervention group in comparison with the control group. Coping style is measured using the Coping Inventory for Stressful Situations (CISS) and the short version of the Social Problem Solving Inventory-Revised (SPSI-R:SF). The CISS questionnaire consists of 48 questions and contains three subscales; Task-oriented coping, Emotion-oriented coping, and Avoidant coping. The subscale Avoidant coping consists of two subscales; Distraction and Social Diversion [18,19]. Because the PST aims at tasks, ‘Task-oriented coping’ is chosen as a primary endpoint; the other two subscales are used as secondary endpoints. The SPSI-R:SF questionnaire consists of ten questions about problem solving skills regarding daily situations. There are five subscales: Positive Problem Orientation, Rational Problem Solving, Negative Problem Orientation, Impulsivity/Carelessness Style, and Avoidance Style, and all are used as secondary endpoints in this trial [20].

HR-QoL is measured using the EuroQol (EQ-5D-5L) and the Stroke Specific Quality of Life Scale (SS-QoL-12). The EQ-5D is a generic questionnaire, and consists of five questions regarding mobility, self-care, daily activities, pain/complaints, mood, and a VAS scale. The five dimensions can be combined to one utility scale, representing the societal perspective of the general public [21]. The SS-QoL-12 is specifically developed for the population of stroke patients [22]. We will use the abbreviated version containing twelve items, which has been shown valid [23]. The questionnaire provides a total score and two sub scores: physical and psychosocial, of which the psychosocial sub-score is defined as the primary endpoint. The other HR-QoL scores are used as secondary endpoints.

Other secondary outcome measures are differences in depression, social participation, and health care consumption between patients in the intervention and control group. Additionally, the influence of cognitive functioning, personality characteristics, aphasia, type of stroke, side of stroke, level of functioning, and demographic characteristics on the outcomes will be assessed. Finally, the cost-effectiveness of the intervention will be calculated compared with standard care.

Depression is measured using the Center for Epidemiologic Studies Depression Scale (CES-D). This questionnaire consists of twenty items concerning depression, higher scores indicate more depressive symptoms [24].

Social participation is measured using the Impact on Participation and Autonomy (IPA). The questionnaire consists of five dimensions; Autonomy indoors, Family role, Autonomy outdoors, Social life and relationships, Work and education [25].

Health care consumption is measured using the Trimbos Questionnaire for Costs association with Psychiatric Illness (TiC-P). The questionnaire was developed for economic evaluation in mental health care, and measures health care consumption and productivity losses [26].

### Sample size calculation

To determine the sample size for measuring differences between the intervention- and control group in coping style and HR-QoL, we searched for comparable effect sizes in the literature. With regard to coping style, there was no data available for this calculation. With regard to HR-QoL, Studenski (2005) measured an increase in HR-QoL after a physical therapy for stroke patients, with a long term effect size f ranging from 0.06 to 0.18 [27]. Because of the lack of more comparable data, we used this data and carefully estimated the effect size f to be 0.08. Considering the design of two groups and four repeated measurements, an expected correlation of 0.70, an alpha of 0.05, and a power of 0.80, we calculated a total required sample size of 132 patients based on the F-test. Because potential drop out is estimated at 0.30, we will strive to include 200 patients.

### Statistical analyses

Demographic variables will be analyzed with an independent sample T-test for continuous variables, the Mann–Whitney U test for ordinal variables, and the chi-square test for categorical variables. Linear Mixed Models will be used to compare the repeated measurements between treatment groups, taking into account the correlation within and between subjects. We will create models for all the primary and secondary outcome variables, with time, group condition (intervention or control), and the interaction between these variables as predictors. Furthermore, we will control for variables that are accidentally not equally distributed between the two group conditions.

The cost-effectiveness of the intervention will be calculated by counting all the medical and non-medical costs, like productivity losses. The incremental cost-effectiveness ratio will be calculated by dividing the difference in total costs by the difference in quality-adjusted life years (QALYs). These QALYs will be calculated based on the EQ-5D questionnaire. The economic evaluation will be conducted according to the Dutch guidelines [28] and includes multivariate probabilistic sensitivity analyses. In the base case scenario the time horizon will be one year. If the effect is still present at one year follow up, a Markov model will be made to model a longer time horizon.

## Discussion

This study investigates the effect of PST on coping style and HR-QoL in stroke patients. In addition, the effect on depression, social participation, and health care consumption will be investigated, as well as the cost-effectiveness of the intervention. We will study the effectiveness of PST as close to the implementation in practice as possible, using a pragmatic trial design and an open group therapy. Any pragmatic trial has limitations; MacPherson (2004) argued that a pragmatic trial design cannot be used to determine the specific components of a treatment that caused an effect [29]. It may be possible that patients in the intervention group show improvement caused by the extra attention they receive and not so much by the assumed effective elements of the therapy; this attention effect may be considered a placebo effect. If we would like to distinguish between this ‘placebo effect’ and the effect of the specific treatment elements, the control group should have received a ‘sham therapy’. Such sham therapy would hinder the estimation of the effect of PST in practice, as in practice such additional effort would not take place. Therefore, one of the advantages of the pragmatic study design is that the external validity is better than using a sham-controlled design; the results will be generalizable to the normal rehabilitation setting [30]. The study population represents the normal stroke population in the outpatient phase of rehabilitation treatment, and the psychologists will provide the intervention to the patients just as they would do in practice. The results of a pragmatic trial are directly applicable to the usual care setting [31]. Moreover, if the intervention will prove effective, it will be easy to implement the intervention in the standard rehabilitation program, since it is already in use and the psychologists will already be trained. Other rehabilitation centers can use the therapy manual we developed.

We expect that patients who received PST will use more effective coping styles and experience a higher HR-QoL. Furthermore, we expect that patients after PST will show a decrease in depression score, an increase in social participation, and a decrease in health care consumption, which would lead to a reduction in the health care costs. We expect the intervention to be cost-effective, since the costs of the intervention are relatively low; one psychologist can train three to six patients at the same time. Darlington (2009) estimated the cost-effectiveness of an intervention aimed at coping strategies in stroke patients: the maximum costs for a single patient were 2500 euros, which will be lower if the therapy is provided in a group [32]. If PST will be proved effective for stroke patients in outpatient rehabilitation, the intervention will be an inexpensive, deliverable and sustainable group intervention that could be added to usual stroke rehabilitation programs.

## Abbreviations

CES-D: Center for Epidemiologic Studies Depression Scale; CISS: Coping Inventory for Stressful Situations; EQ-5D: EuroQol-5D; HR-QoL: Health-related quality of life; IPA: Impact on Participation and Autonomy; PST: Problem Solving Therapy; QALYs: Quality-adjusted life years; RCT: Randomized controlled trial; SPSI-R: Social Problem Solving Inventory-Revised; SS-QoL: Stroke Specific Quality of Life Scale; TBI: Traumatic brain-injured; TiC-P: Trimbos Questionnaire for Costs association with Psychiatric Illness; WHOQOL: World Health Organization Quality of Life.

## Competing interests

The authors declare that they have no competing interests.

## Authors’ contributions

All authors contributed to the development of the study protocol. AvtS adapted the PST-manual for the population of stroke patients. MMV implemented the therapy in the rehabilitation program. All authors read and corrected the draft version of this manuscript, and approved the final version.

## Pre-publication history

The pre-publication history for this paper can be accessed here:

http://www.biomedcentral.com/1471-2377/13/67/prepub
